# Supplementary material for: Meta-Analysis and Gene Set Enrichment Relative to ER Status Reveal Elevated Activity of MYC and E2F in the “Basal” Breast Cancer Subgroup
Source: PLoS One. 2009 Mar 9;4(3):e4710. doi: 10.1371/journal.pone.0004710 (PMC2650420; doi:10.1371/journal.pone.0004710)
Supplement: Dataset S1 — GSEA results from first screen. The file contains a zipped archive of the HTML results of the second GSEA screen. Reports for enriched or depleted datasets may be accessed from the “index.html” link within the my_analysis.GseaPreranked.1219975950625.rpt.zip ZIP archive. Enrichment plots are available for each of the 50 most enriched and depleted gene sets. (13.32 MB ZIP) [file pone.0004710.s001.zip › my_analysis.GseaPreranked.1219975950625.rpt.zip!//BRCA_PROGNOSIS_POS.html]

Details for gene set BRCA\_PROGNOSIS\_POS[GSEA]

|  || Dataset | zSco.ERneg.to.ERpos\_collapsed |
| Phenotype | NoPhenotypeAvailable |
| Upregulated in class | na\_neg |
| GeneSet | BRCA\_PROGNOSIS\_POS |
| Enrichment Score (ES) | -0.7971276 |
| Normalized Enrichment Score (NES) | -2.9900892 |
| Nominal p-value | 0.0 |
| FDR q-value | 0.0 |
| FWER p-Value | 0.0 |
Table: GSEA Results Summary

  

Fig 1: Enrichment plot: BRCA\_PROGNOSIS\_POS      
 Profile of the Running ES Score & Positions of GeneSet Members on the Rank Ordered List

  

| PROBE | GENE SYMBOL | GENE\_TITLE | RANK IN GENE LIST | RANK METRIC SCORE | RUNNING ES | CORE ENRICHMENT || 1 | KIAA1217 |  |  | 3218 | 1.829 | -0.2269 | No |
| 2 | RBP3 |  |  | 3444 | 1.733 | -0.2292 | No |
| 3 | PDIA4 |  |  | 3785 | 1.592 | -0.2414 | No |
| 4 | FGF18 |  |  | 4851 | 1.206 | -0.3114 | No |
| 5 | PCSK6 |  |  | 5798 | 0.878 | -0.3753 | No |
| 6 | ALDH6A1 |  |  | 6317 | 0.711 | -0.4083 | No |
| 7 | WISP1 |  |  | 6853 | 0.534 | -0.4441 | No |
| 8 | RPS4X |  |  | 7738 | 0.218 | -0.5088 | No |
| 9 | NEO1 |  |  | 9529 | -0.577 | -0.6387 | No |
| 10 | CHPT1 |  |  | 9702 | -0.670 | -0.6460 | No |
| 11 | BBC3 |  |  | 9752 | -0.695 | -0.6438 | No |
| 12 | ALDH4A1 |  |  | 10425 | -1.102 | -0.6852 | No |
| 13 | ACADS |  |  | 11005 | -1.571 | -0.7155 | No |
| 14 | C20ORF28 |  |  | 12090 | -2.693 | -0.7745 | Yes |
| 15 | KIAA1324 |  |  | 12142 | -2.762 | -0.7551 | Yes |
| 16 | EVL |  |  | 12166 | -2.798 | -0.7332 | Yes |
| 17 | MYLIP |  |  | 12172 | -2.805 | -0.7100 | Yes |
| 18 | QDPR |  |  | 12196 | -2.838 | -0.6878 | Yes |
| 19 | MATN3 |  |  | 12441 | -3.165 | -0.6796 | Yes |
| 20 | LETMD1 |  |  | 12467 | -3.211 | -0.6544 | Yes |
| 21 | C20ORF103 |  |  | 12514 | -3.286 | -0.6302 | Yes |
| 22 | PEX12 |  |  | 12555 | -3.341 | -0.6051 | Yes |
| 23 | RAB27B |  |  | 12576 | -3.378 | -0.5782 | Yes |
| 24 | AP2B1 |  |  | 12670 | -3.557 | -0.5553 | Yes |
| 25 | TGFB3 |  |  | 12734 | -3.702 | -0.5288 | Yes |
| 26 | TBX3 |  |  | 12795 | -3.880 | -0.5007 | Yes |
| 27 | FBP1 |  |  | 12849 | -4.003 | -0.4710 | Yes |
| 28 | STK32B |  |  | 13030 | -4.556 | -0.4462 | Yes |
| 29 | BTG2 |  |  | 13039 | -4.613 | -0.4080 | Yes |
| 30 | KIF3B |  |  | 13084 | -4.771 | -0.3711 | Yes |
| 31 | PIB5PA |  |  | 13125 | -4.921 | -0.3327 | Yes |
| 32 | FUT8 |  |  | 13195 | -5.349 | -0.2929 | Yes |
| 33 | PECI |  |  | 13201 | -5.385 | -0.2479 | Yes |
| 34 | CIRBP |  |  | 13206 | -5.436 | -0.2025 | Yes |
| 35 | TBC1D9 |  |  | 13226 | -5.639 | -0.1564 | Yes |
| 36 | SCUBE2 |  |  | 13239 | -5.755 | -0.1089 | Yes |
| 37 | GSTM3 |  |  | 13252 | -5.994 | -0.0593 | Yes |
| 38 | KRT18 |  |  | 13317 | -7.649 | 0.0002 | Yes |
Table: GSEA details [plain text format]

  

Fig 2: BRCA\_PROGNOSIS\_POS: Random ES distribution      
 Gene set null distribution of ES for **BRCA\_PROGNOSIS\_POS**
